# Supplementary material for: Hot spot prediction in protein-protein interactions by an ensemble system
Source: BMC Syst Biol. 2018 Dec 31;12(Suppl 9):132. doi: 10.1186/s12918-018-0665-8 (PMC6311905; doi:10.1186/s12918-018-0665-8)
Supplement: Supplementary file 2 — All relevant MATLAB code. A simple Matlab implement of our predictor. (ZIP 19,742 kb) [file 12918_2018_665_MOESM2_ESM.zip › Additional file 2/Read ASA/ASA Property/SKEMPI/1R0RI.txt]

 NetSurfP - Protein Surface Accessibility and Secondary Structure Predictions

  

|  |  |  |
| --- | --- | --- |
|  |  | NetSurfP - Protein Surface Accessibility and Secondary Structure Predictions  Technical University of Denmark |

  


---

```
# For publication of results, please cite:
# A generic method for assignment of reliability scores applied to solvent accessibility predictions.
# Bent Petersen, Thomas Nordahl Petersen, Pernille Andersen, Morten Nielsen and Claus Lundegaard
# BMC Structural Biology 2009, 9:51 doi:10.1186/1472-6807-9-51
#
# Column 1: Class assignment - B for buried or E for Exposed - Threshold: 25% exposure, but not based on RSA
# Column 2: Amino acid
# Column 3: Sequence name
# Column 4: Amino acid number
# Column 5: Relative Surface Accessibility - RSA
# Column 6: Absolute Surface Accessibility
# Column 7: Z-fit score for RSA prediction
# Column 8: Probability for Alpha-Helix
# Column 9: Probability for Beta-strand
# Column 10: Probability for Coil
E V  1R0RI                  1    0.576  88.470  -1.434   0.005   0.015   0.979
E D  1R0RI                  2    0.636  91.590   0.106   0.004   0.138   0.858
B C  1R0RI                  3    0.159  22.380  -1.665   0.018   0.047   0.935
E S  1R0RI                  4    0.658  77.106   0.642   0.053   0.043   0.903
E E  1R0RI                  5    0.617 107.860   1.058   0.053   0.043   0.903
B Y  1R0RI                  6    0.199  42.612   0.734   0.018   0.047   0.935
E P  1R0RI                  7    0.568  80.642   0.425   0.018   0.088   0.893
E K  1R0RI                  8    0.600 123.379   0.108   0.019   0.141   0.840
E P  1R0RI                  9    0.313  44.401  -0.107   0.019   0.141   0.840
E A  1R0RI                 10    0.328  36.102  -0.566   0.018   0.088   0.893
B C  1R0RI                 11    0.050   7.034  -0.227   0.018   0.047   0.935
E T  1R0RI                 12    0.340  47.116  -0.276   0.018   0.047   0.935
E L  1R0RI                 13    0.422  77.323  -0.640   0.052   0.084   0.864
E E  1R0RI                 14    0.449  78.493  -0.533   0.056   0.142   0.802
B Y  1R0RI                 15    0.219  46.779  -0.111   0.056   0.142   0.802
E R  1R0RI                 16    0.426  97.508  -0.331   0.052   0.084   0.864
B P  1R0RI                 17    0.126  17.823  -0.668   0.052   0.084   0.864
B L  1R0RI                 18    0.102  18.676   0.154   0.064   0.216   0.721
B C  1R0RI                 19    0.058   8.143   0.106   0.066   0.296   0.638
B G  1R0RI                 20    0.165  12.986  -1.203   0.056   0.142   0.802
E S  1R0RI                 21    0.397  46.564  -0.936   0.018   0.047   0.935
E D  1R0RI                 22    0.496  71.502  -0.572   0.018   0.019   0.964
E N  1R0RI                 23    0.400  58.633  -1.725   0.018   0.019   0.964
E K  1R0RI                 24    0.380  78.084   0.258   0.018   0.088   0.893
B T  1R0RI                 25    0.287  39.876  -0.662   0.020   0.205   0.775
B Y  1R0RI                 26    0.157  33.444   0.616   0.118   0.150   0.732
E G  1R0RI                 27    0.541  42.569  -0.881   0.181   0.016   0.803
B N  1R0RI                 28    0.224  32.838  -1.008   0.430   0.016   0.555
E K  1R0RI                 29    0.361  74.196   0.511   0.751   0.050   0.199
B C  1R0RI                 30    0.047   6.599   0.517   0.779   0.100   0.120
E N  1R0RI                 31    0.299  43.715   0.843   0.779   0.100   0.120
B F  1R0RI                 32    0.095  19.087   0.656   0.870   0.077   0.053
B C  1R0RI                 33    0.077  10.797  -0.199   0.870   0.077   0.053
B N  1R0RI                 34    0.281  41.124   0.316   0.870   0.077   0.053
E A  1R0RI                 35    0.294  32.366  -0.390   0.807   0.137   0.056
B V  1R0RI                 36    0.252  38.656   0.491   0.746   0.197   0.057
B V  1R0RI                 37    0.194  29.879   0.490   0.662   0.225   0.113
E E  1R0RI                 38    0.558  97.465  -0.016   0.453   0.248   0.299
E S  1R0RI                 39    0.541  63.440  -1.182   0.113   0.087   0.800
E N  1R0RI                 40    0.681  99.654  -0.322   0.018   0.019   0.964
E G  1R0RI                 41    0.330  25.979  -0.693   0.005   0.045   0.951
E T  1R0RI                 42    0.474  65.716   1.303   0.005   0.336   0.660
B L  1R0RI                 43    0.039   7.232  -0.061   0.002   0.816   0.182
E T  1R0RI                 44    0.319  44.204   1.087   0.001   0.900   0.099
B L  1R0RI                 45    0.088  16.076   1.056   0.001   0.959   0.040
E S  1R0RI                 46    0.387  45.403   1.255   0.001   0.959   0.040
B H  1R0RI                 47    0.267  48.622  -0.131   0.001   0.900   0.099
E F  1R0RI                 48    0.509 102.096  -0.086   0.004   0.514   0.481
E G  1R0RI                 49    0.306  24.066  -2.634   0.005   0.336   0.660
E K  1R0RI                 50    0.668 137.325   1.218   0.005   0.262   0.733
E C  1R0RI                 51    0.517  72.517  -2.014   0.003   0.003   0.994

Explain the output.  Go back.
```

---
